# Evaluation of pregnancy outcomes from the Tysabri® (natalizumab) pregnancy exposure registry: a global, observational, follow-up study

**DOI:** 10.1186/s12883-016-0674-4

**Published:** 2016-08-24

**Authors:** Susan Friend, Sandra Richman, Gary Bloomgren, Lynda M. Cristiano, Madé Wenten

**Affiliations:** 1 Biogen, Innovation House, 70 Norden Road, Maidenhead, Berkshire SL6 4AY UK; 2 Biogen, Cambridge, MA USA

**Keywords:** Pregnancy outcome, Multiple sclerosis, Fetal development, Spontaneous abortion, Live birth, Follow-up studies

## Abstract

**Background:**

Patients with multiple sclerosis (MS) or Crohn’s disease (CD) being treated with natalizumab (Tysabri®, Biogen) who are planning to become pregnant or discover they are pregnant after exposure to natalizumab are currently advised to balance the potential benefits and potential risks of exposure when considering treatment options. This study was undertaken to evaluate pregnancy outcomes of women with MS or CD who were exposed to natalizumab at any time within 3 months prior to conception or during pregnancy. A pregnancy registry was created to better understand the effect of natalizumab exposure on pregnancy outcomes.

**Methods:**

The Tysabri Pregnancy Exposure Registry was a global, observational exposure registration and follow-up study. Evaluations included spontaneous abortions (<22 weeks gestational age), fetal losses (≥22 weeks gestational age), ectopic pregnancies, elective or therapeutic terminations, stillbirths, birth defects, and live births. Birth defects were reviewed and coded in accordance with the Metropolitan Atlanta Congenital Defects Program (MACDP) classification of birth defects.

**Results:**

A total of 369 patients with MS and 7 patients with CD were enrolled prospectively, of whom 355 patients (99.4 %; 349 MS and 6 CD) had known pregnancy outcomes (including 8 twin sets). The spontaneous abortion rate was 9.0 % (*n =* 32; 95 % confidence interval [C. I.], 6.3–12.5 %). An independent advisory committee review determined the major birth defect rate to be 5.05 % (16 of 316 live births + 1 elective abortion; 95 % C. I., 2.9–8.1 %). The mean gestational age of the live-born infants was 38.3 weeks, and the mean birth weight was 3158.3 g.

**Conclusions:**

Although the overall rate of birth defects was higher than that observed by the MACDP, these registry outcomes showed no specific pattern of malformations that would suggest a drug effect, and the spontaneous abortion rate was consistent with that of the general population.

**Trial registration:**

ClinicalTrials.gov NCT00472992 (11 May 2007).

## Background

Women of childbearing potential comprise a considerable segment of the patient population affected by multiple sclerosis (MS) and Crohn’s disease (CD) and may be exposed to therapies around conception and during pregnancy. Two-thirds of patients with MS are women, with a peak onset between 20 and 34 years of age, and approximately 10 % have disease onset during pregnancy [1–3]. CD peak onset is between 15 and 35 years of age, and in some regions there is a slight female predominance (20–30 % more frequently in women), particularly in high-incidence areas [4]. Women with MS or CD should be counseled to balance the benefits and risks of exposure when considering treatment options before or during pregnancy [5].

Natalizumab (Tysabri®; Biogen, Cambridge, MA, USA) is a humanized monoclonal antibody indicated for relapsing-remitting MS (RRMS) [6] that prevents leukocyte migration into the brain and reduces inflammation in MS patients [7, 8]. Within the United States, natalizumab is also approved for treatment of patients with CD [6]; in CD, natalizumab inhibits leukocyte adhesion and migration into gut tissue [9]. Studies of natalizumab in MS have shown reduced relapse rates and disability progression, but less is known about its effects on pregnancy outcomes [10, 11]. Natalizumab is classified as a pregnancy category C drug, as potential fetal effects have been reported in animal studies [12–14] and there is a paucity of well-controlled human studies [6]. Although some animal studies have shown that natalizumab can cross the placental barrier and produce hematologic effects on fetal guinea pigs and primates [6, 12, 14, 15], others have not shown fetal interaction [16]. Human studies and case reports have not shown increases in spontaneous abortions or birth defects; however, results are limited by small sample sizes [17–20].

This study prospectively evaluated pregnancy outcomes of women with MS or CD who were exposed to natalizumab within the 3 months before conception or during pregnancy.

## Methods

### Study design

The Tysbari Pregnancy Exposure Registry (TPER; referred to as the Registry) was a global, observational, exposure registration and follow-up study of pregnant women with MS or CD. The Registry collected information routinely documented in the patient and infant medical record with no Registry-required interventions or procedures conducted.

### Ethics, consent, and permissions

The Registry (ClinicalTrials.gov NCT00472992) was established in accordance with regulatory guidance for pregnancy registries [21–23]. Independent ethics committees (Aspire Institutional Review Board, Santee, CA, USA, and Institutional Review Board Services, Auroria, ON, Canada) reviewed and approved this Registry protocol. This study was conducted in accordance with the ethical principles of Good Clinical Practice based on the International Conference on Harmonisation Harmonised Tripartite Guideline. All patients provided written informed consent and were free to withdraw participation in the Registry at any time.

### Patients

Between February 14, 2007, and April 24, 2011, women with MS or CD who were exposed to natalizumab at any time within the 3 months prior to conception or during pregnancy and for whom the outcome of the pregnancy was unknown at the time of enrollment were eligible to be registered prospectively in the study. Patients included women enrolled from observational studies (TYGRIS [Tysabri Global Observational Program in Safety; NCT00477113 and NCT00483847] or CD INFORM [Crohn’s Disease Investigating Natalizumab through Further Observational Research and Monitoring; NCT00707512]) or who received natalizumab as a marketed product in the United States and the rest of the world (ROW). The Coordinating Center (CC) of the Registry monitored patients throughout pregnancy and monitored outcomes within 4 weeks after the estimated date of delivery (United States and ROW) and within 8–12 weeks post delivery (United States). The CC point-of-contact and schedule varied slightly based on the study setting.

### Registry data collection

Information was collected about natalizumab exposure, potential confounding factors (e.g., medical history, concomitant medications, or smoking), pregnancy outcomes, spontaneous abortions, fetal losses including stillbirths, and ectopic pregnancies. In addition, data were collected for elective or therapeutic pregnancy terminations, live-born infants, and birth defects.

Birth defects were reviewed and coded by an independent birth defect evaluator (a specialist in pediatrics and genetics) in accordance with the Metropolitan Atlanta Congenital Defects Program (MACDP) classification of birth defects [24]. A major birth defect was defined as one characterized by a major structural or chromosomal abnormality in any live or stillborn infant or electively terminated fetus; any other birth defect was classified as minor. The MACDP excludes birth defects that are attributable to prematurity alone or identified prior to 20 weeks’ gestation. At the end of the study, an independent scientific advisory committee, consisting of 3 experts in relevant specialties of teratology, epidemiology, and maternal and fetal medicine, evaluated all Registry outcomes.

### Statistical analysis

A target sample size of 300 pregnancy outcomes was determined based on the ability to detect a 2-fold increase in spontaneous abortion and a 3-fold increase in fetal loss and any major birth defects over general population background rates with 80 % power at the 0.05 level of significance.

For the primary analysis, the major birth defect rate was calculated by dividing the number of infants with major birth defects by the total number of live births. Birth defect rates from the Registry were compared with available background rates from the MACDP in the US general population [25]. A further calculation of the birth defect rate was performed excluding those birth defects that were considered to be non-major defects by the MACDP [25]. The rate of spontaneous abortions was calculated by dividing the number of fetal losses at <22 weeks gestational age by the total number of pregnancies. Corresponding 95 % confidence intervals (C. I.s) were calculated for major birth defect and spontaneous abortion rates. Spontaneous abortion rates in the Registry were compared with general population rates reported in published literature [26–28].

## Results

### Patients

**Fig. 1:**
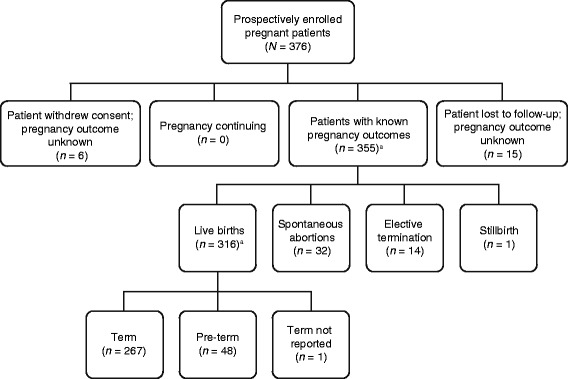
Patients enrolled and outcome of pregnancy. ^a^Eight completed pregnancies resulted in twin births

The first patient was enrolled on February 14, 2007, and the last pregnancy outcome was obtained on April 24, 2012. A total of 376 patients were prospectively enrolled in the Registry, with 318 patients enrolled in the United States (TYGRIS, *n =* 19; CD INFORM, *n =* 2; non-study, *n =* 297) and 58 enrolled in the ROW (TYGRIS, *n =* 58). The majority of patients had MS; only 7 of 376 patients had CD. Of the 376 patients, 6 (1.6 %) withdrew consent and 15 (4.0 %) were lost to follow-up; 355 (94.4 %) patients (including 8 sets of twins) had known pregnancy outcomes, resulting in a total of 363 known outcomes (Fig. 1).

The mean age of the study population was 30.4 years (standard deviation [SD], 5.15 years; range 16–45 years). Race was only collected in patients enrolled in the United States (*N =* 318). Of US patients enrolled, 73.3 % were White, 16.4 % were Black, 6.6 % were Hispanic, and 0.9 % were Asian. Of the 9 patients (2.8 %) listed as “other”, 3 identified as biracial and 1 patient each as European, Multi, Filipino, Persian, Middle Eastern, and unknown.

### Maternal obstetric history and prenatal natalizumab use

The maternal characteristics of patients at enrollment are detailed in Table 1. More than half of the patients (58.8 %) had experienced ≥1 prior pregnancy. Reports of prior birth defects were rare; 2 patients reported prior offspring with birth defects/mental handicap. The mean gestational age at the time of enrollment was 11.8 weeks (range 3–39 weeks).

**Table 1:** Baseline characteristics of study population (*N =* 376)

Maternal characteristic	*n* (%)
Age	
≤ 20 years	10 (2.7)
> 20 to 30 years	181 (48.1)
> 30 to 40 years	178 (47.3)
> 40 to 50 years	7 (1.9)
Obstetric history	
≥ 1 prior pregnancy	221 (58.8)
1 prior pregnancy resulting in fetal stillbirth (≥ 22 weeks)	2 (0.5)
1 prior miscarriage	53 (14.1)
2 prior miscarriages	11 (2.9)
3 prior miscarriages	4 (1.1)
4 prior miscarriages	1 (0.3)
> 4 prior miscarriages	2 (0.5)
> 4 prior pregnancies resulting in fetal stillbirth (≥ 22 weeks)	1 (0.3)
1 prior elective/therapeutic abortion	28 (7.4)
2 prior elective/therapeutic abortions	4 (1.1)
1 prior ectopic pregnancy	3 (0.8)
Concomitant medications	
Prenatal vitamins	168 (44.7)
Baclofen	29 (7.7)
Folic acid	22 (5.9)
Modafinil	21 (5.6)
Multivitamins	19 (5.1)
Prior medical history	
Chicken pox	294 (78.2)
Urinary tract infection	135 (35.9)
Abnormal Pap test	109 (29.0)
Gynecological surgery	55 (14.6)
Breathing disorder	46 (12.2)
Sexually transmitted disease	39 (10.4)
Breast cancer	1 (0.3)
Cervical cancer	1 (0.3)
Lymphoma	1 (0.3)
Melanoma	1 (0.3)
Social risk factors^a^	
Caffeine use	235 (62.5)
Alcohol consumption	70 (18.6)
Tobacco use	59 (15.7)
Illicit drug use	7 (1.2)

^a^Of patients who reported caffeine, alcohol, tobacco, or illicit drug use at enrollment, 168 of 235 (71.5 %), 7 of 70 (10.0 %), 25 of 59 (42.4 %), and 1 of 7 (14.3 %), respectively, continued use during pregnancy

Most enrolled patients (*n =* 366; 97.3 %) had discontinued natalizumab use at the time of enrollment. Of the 376 patients enrolled, 71 (18.9 %) discontinued natalizumab during the 3-month period prior to conception, 288 (76.6 %) discontinued during the first trimester, and 7 (1.9 %) discontinued during the second trimester. Six patients discontinued natalizumab after enrollment: 2, 3, and 1 patients during the first, second, and third trimesters, respectively.

### Pregnancy outcomes

#### Birth defects

Of the 363 pregnancy outcomes in 355 patients, 57 birth defects (minor or major) were confirmed in 30 infants (including 3 sets of twins) (Tables 2 and 3); of these, 29 were live births and 1 was an elective termination resulting from a birth defect. In the case of the elective termination, natalizumab exposure was during the 3 months prior to conception. Of the 29 live births with birth defects, all were born to mothers who were last exposed to natalizumab during preconception or in the first trimester. More than half of these infants (*n =* 17, 58.6 %) were last exposed between gestational weeks 1 and 4, while the remainder were last exposed within 90 days prior to the last menstrual period (*n =* 6, 20.7 %), between 5 and 8 weeks’ gestation (*n =* 5, 17.2 %), or between 9 and 13 weeks’ gestation (*n =* 1, 3.4 %). The mean age of mothers who had infants with birth defects was 31.8 years (range 24–45 years).

**Table 2:** Pregnancy outcome categorized by time of discontinuation of natalizumab

Outcome of pregnancy, n (%)	Before conception (*n =* 73)	First trimester (*n =* 275)	Second trimester (*n =* 10)	Third trimester (*n =* 1)	Continued during pregnancy (*n =* 4)	Total (*N =* 363)
Spontaneous abortions	0	32 (11.6)	0	0	0	32 (8.8)
Elective termination (fetal defects)	1 (1.4)	0	0	0	0	1 (0.3)
Elective termination (no fetal defects or unknown)	2 (2.7)	11 (4.0)	0	0	0	13 (3.6)
Stillbirths without fetal defects	1 (1.4)	0	0	0	0	1 (0.3)
Live birth with congenital anomaly	7 (9.6)^a^	22 (8.0)^b^	0	0	0	29 (8.0)
Live birth without congenital anomaly	62 (84.9)^c^	210 (76.4)^a^	10 (100)	1 (100)	4 (100)	287 (79.1)

^a^Includes 2 sets of twins

^b^Includes 1 set of twins

^c^Includes 3 sets of twins

**Table 3:** All observed major and minor birth defects (per MACDP criteria [25]) by organ system

Organ system, birth defect	Major or minor birth defect	Number of events (*n =* 57)
Musculoskeletal (*n =* 11)		
Plagiocephaly^a^	Minor	4
Torticollis^a^	Major	4
Hip dysplasia	Major	1
Polydactyly	Major	1
Absent right femur	Major	1
Cardiovascular (*n =* 10)		
Ventricular septal defect^b^	Major	3
Patent foramen ovale^c^	Minor	2
Atrial shunt	Major	1
Patent ductus arteriosus^c^	Minor	1
Supraventricular tachycardia	Minor	1
Tetralogy of Fallot	Major	1
Tricuspid valve atresia	Major	1
Skin (*n =* 9)		
Hemangioma	Minor	2
Café-au-lait spot	Minor	1
Irregular tragus	Minor	1
Mongolian spot	Minor	1
Small anterior fontanelle	Minor	1
Subcutaneous cyst	Minor	1
Unspecified anomaly of nose	Major	1
Unspecified anomaly of toes	Minor	1
Genital disorders (*n =* 8)		
Chordee	Minor	2
Hydrocele	Minor	2
Undescended testicle	Minor	2
Chordee with hypospadias	Major	1
Penile concealment	Major	1
Neurologic (*n =* 6)		
Colpocephaly	Major	1
Holoprosencephaly	Major	1
Hydrocephalus	Major	1
Myelomeningocele	Major	1
Sacral dimple	Minor	1
Unspecified agenesis of corpus callosum	Major	1
Renal (*n =* 5)		
Hydronephrosis	Major	2
Cystic dysplasia	Major	1
Renal dilation	Minor	1
Vesicoureteral reflux	Major	1
Gastrointestinal (*n =* 4)		
Umbilical hernia	Minor	2
Inguinal hernia	Minor	1
Tight frenulum	Minor	1
Chromosome (*n =* 1)		
Partial trisomy 9^d^	Major	1
Endocrine (*n =* 1)		
Congenital hypothyroidism	Minor	1
Metabolic (*n =* 1)		
Phenylketonuria	Major	1
Ocular (*n =* 1)		
Disconjugate gaze	Major	1

^a^Three cases of plagiocephaly and 2 cases of torticollis occurred in 2 sets of twins

^b^No cases occurred in a premature birth (i.e., <37 weeks)

^c^One case occurred in a premature birth (35 weeks 4 days’ gestation)

^d^Maternal age was 46 years at enrollment

Of the 57 observed birth defects (Table 3), those characterized as a major structural or chromosomal abnormality in a live or stillborn infant or electively terminated fetus were identified in 25 infants/fetuses out of 317 cases (316 live births plus 1 elective termination), leading to an overall rate of major structural or chromosomal abnormality of 7.9 % (95 % C. I. 5.2–11.4 %). To facilitate comparison of Registry data with the birth defect rate calculated by MACDP, a further calculation of the major birth defect rate was performed to exclude those birth defects not considered to be major by the MACDP [25]. This resulted in 18 of 317 infants with major birth defects, representing a rate of 5.7 % (95 % C. I. 3.4–8.8 %). Finally, the Advisory Committee reviewed all available Registry data; of the 18 cases identified as major birth defects using MACDP criteria, 3 cases of torticollis were considered medically minor and were excluded. However, 1 case of congenital hypothyroidism, which had been excluded by MACDP criteria, was considered by the Committee to be a major defect and was included. Therefore, the final adjusted rate of major birth defects, as adjudicated by the Advisory Committee, was determined in 16 of 317 cases, or 5.05 % (95 % C. I. 2.90–8.11 %).

#### Birth defects with possible temporal relationship

A birth defect evaluator also assessed confirmed defects (minor and major) for a possible temporal relationship to natalizumab exposure. A total of 26 major and minor defects were judged to have a possible temporal relationship (i.e., the development of the defect and the timing of natalizumab exposure could not rule out a possible association), with 16 major defects in 12 infants and 10 minor defects in 7 infants (Table 4).

**Table 4:** Major and minor birth defects with possible temporal relationship^a^ to exposure

Major birth defects	Minor birth defects	Gestational age at last natalizumab dose	Maternal concomitant medications
Absent right femur	NA	4 weeks	Azelastine nasal, prenatal vitamins, zolpidem
Anomaly of nose, cystic dysplasia of kidney, holoprosencephaly	Sandal toe gap, small anterior fontanelle	3 weeks	Methylphenidate, prenatal vitamins
Atrial shunt	NA	1 week	Heparin sodium, prenatal vitamins
Chordee with hypospadias	NA	26 days	Fluvoxamine, hydrocodone/acetaminophen, lamotrigine, meperidine/promethazine, ondansetron hydrochloride, prenatal vitamins
Colpocephaly, hydrocephalus, partial agenesis of corpus callosum	NA	4 days	Methylprednisolone sodium succinate, sertraline hydrochloride
Hydronephrosis	NA	5 weeks	Levothyroxine, metformin, sertraline hydrochloride
Penile concealment	NA	3 weeks	Prenatal vitamins
Polydactyly	NA	5 days	Folic acid, insulin
Tetralogy of Fallot	NA	0 (29 days prior to LMP)	Duloxetine, prenatal vitamins, topiramate, trazodone
Torticollis	NA	8 weeks	Bupropion, methylprednisolone, pantoprazole, propoxyphene/ acetaminophen, topiramate
Tricuspid valve atresia	NA	3 weeks	NR
Ventricular septal defect	NA	1 day	NR
NA	Congenital hypothyroidism	4.5 weeks	Nitrofurantoin, prenatal vitamins, Rh_0_(D) immune globulin, venlafaxine hydrochloride
NA	Irregular tragus, umbilical hernia	11 weeks	Prenatal vitamins
NA	Mongolian spot	3 weeks	Escitalopram, hydrocodone, levothyroxine, modafinil, prenatal vitamins
NA	Sacral dimple, umbilical hernia	5 weeks	Prenatal vitamins, valacyclovir
NA	Subcutaneous cyst	3 weeks	Prenatal vitamins
NA	Tight frenulum	3 days	Prenatal vitamins, tizanidine

*Abbreviations*: *LMP* last menstrual period, *NA* not applicable, *NR* not reported

^**a**^Potential relevance of the timing of natalizumab exposure to the birth defect(s) was evaluated and coded by the geneticist as “known cause”, “unknown cause”, “no association”, or “possible association”

#### Spontaneous abortions and fetal demise

Among the 355 pregnancies resulting in 363 known pregnancy outcomes, mean gestational age at enrollment was 11.8 weeks (SD, 6.8 weeks; range 3–39 weeks). In the 32 spontaneous abortions observed, the mean gestational age at enrollment was 5.5 weeks (SD, 2.0 weeks; range 4–13 weeks). Thus, the rate of spontaneous abortion (defined as fetal loss prior to 22 weeks’ gestation) among pregnancies with known outcomes was 9.0 % (95 % C. I. 6.3–12.5 %). Of the 355 pregnancies with known outcomes, 339 were enrolled prior to 22 weeks’ gestation; the rate of spontaneous abortion among these 339 pregnancies was 9.4 % (95 % C. I. 6.6–13.1 %). One stillbirth (0.3 %) was reported, and 14 patients (3.9 %) had elective terminations, 1 with a birth defect. No ectopic pregnancies were reported.

#### Physical attributes of live born infants

Of the 316 live born infants, 287 (90.8 %) were born without congenital anomaly. There were 148 males (46.8 %) and 163 females (51.6 %); gender was not recorded for 5 infants (1.6 %). The mean gestational age at birth was 38.3 weeks (range 26–43 weeks). The majority of infants (267 of 316, 84.5 %) were born at term (≥37 weeks); 48 (15.2 %) were born prematurely, and gestational age at birth was not recorded for 1 infant (0.3 %). At birth, mean APGAR (appearance, pulse, grimace, activity, and respiration) scores were 8.0 (range 1–10) at 1 min, 8.9 (range 2–10) at 5 min, and 9.6 (range 6–10) at 10 min; mean birth weight was 3161.7 g (95 % C. I. 3099.5–3223.9 g), mean infant length was 49.7 cm (range 34–56 cm), and mean head circumference was 35.2 cm (range 30–89 cm). Of the 290 singleton births, 22 (7.6 %) resulted in low birth weight, which compares favorably with that reported by the US National Center for Health Statistics (6.27 %) for the 2013 rate of low birth weight in singleton births [29].

## Discussion

Currently, women with MS or CD being treated with natalizumab who are planning to become pregnant or discover they are pregnant after natalizumab exposure are advised to balance the potential benefits and potential risks of exposure when considering treatment options. Although some patients are able to discontinue treatment before or during pregnancy, others with more severe disease may elect to continue treatment. Thus, this Registry was undertaken to prospectively gather important information about pregnancy outcomes in patients exposed to natalizumab. In the Registry, the rate of reported birth defects in the infants of women exposed to natalizumab during pregnancy was higher than the 2.67 % observed in the MACDP. No specific pattern of malformations was seen within the observed birth defects. The rate of spontaneous abortions was consistent with the general population [26–28].

Natalizumab prevents leukocyte migration by binding to α4 integrins that are expressed on the surface of leukocytes [6]. However, reproductive and/or fetal development processes may be affected by inhibition of α4 integrins; of particular note are the processes of fertilization, placental development, embryo implantation, hematopoiesis, and cardiac development [30–35]. Natalizumab has been categorized as a pregnancy category C medication based on animal studies showing transplacental crossing and potential for offspring effects, such as reduced pup survival in guinea pigs and mild anemia or reduced platelet count in the fetus of monkeys [6, 12, 13]. However, no treatment-related teratogenic effects were observed, including no cardiac abnormalities observed in any natalizumab study, in contrast to the published role of α4 in the formation of the epicardium and consequent cardiac abnormalities in α4 null mice [12, 16, 34].

Animal studies provide valuable information, but human studies are also necessary to understand maternal and neonatal outcomes. Hellwig et al reported no decreased fetal growth or teratogenicity in 35 patients who received natalizumab for ≥8 weeks prior to their last menses and discontinued treatment as soon as they became aware of the pregnancy [17]. In these patients, the rate of spontaneous abortions (14.3 % [5 of 35]) was higher than that observed in the Registry (9.0 %). Of the remaining 30 patients in their study, 1 underwent an elective termination, and 29 women birthed 28 healthy newborns and 1 infant with a hexadactyly defect that was also captured in this Registry. The average birth weight in the natalizumab group (3159 g) was within normal range for full-term infants (i.e., 2500–4000 g [36]), although slightly lower than that in the group of pregnant patients with MS who had not been exposed to disease modifying therapies (3406 g). Additional case reports by Mattioda et al [19], Totaro et al [20], and Hoevenaren et al. [18] did not show any abnormalities in infants at birth and at a 6-week follow-up.

The potential influence of natalizumab exposure at different gestational time points should be considered. Case reports by Mattioda et al [19] and Totaro et al. [20] found that first-trimester exposure to natalizumab resulted in uncomplicated gestation and neonatal outcomes (normal fetal growth and full-term delivery). Houtchens et al reported on 2 patients with natalizumab exposure during the first 6 weeks of pregnancy, resulting in 1 healthy infant and 1 miscarriage [15]. Fagius and Burman reported on a patient with MS who continued natalizumab treatment throughout the pregnancy, resulting in an uncomplicated caesarian delivery at full-term and a normal infant at an 8-month follow-up [37]. Although few third-trimester exposures to natalizumab were reported in the Registry, a recent case series of 13 pregnancies in women with aggressive MS observed hematological abnormalities in 10 of 13 newborns following third-trimester exposure to natalizumab [38].

In the Registry, the majority of patients discontinued treatment prior to or within the first trimester. The pregnancies resulting in live births with defects were last exposed to natalizumab within 3 months prior to conception or within the first trimester, with more than half exposed between 1 and 4 weeks of gestation. Of the 4 patients who continued natalizumab treatment during pregnancy, no congenital anomalies were observed in the resulting 4 live births. Interpretation of the Registry data is limited by the duration of natalizumab exposure during pregnancy.

The Registry found no increased risk of pregnancy loss in pregnancy outcomes among women with natalizumab exposure compared with rates in the general population. The spontaneous abortion rate of patients in the Registry (9.0 %) was lower than that observed in the general population (13.1–15.9 %) [26, 27] and consistent with a longitudinal study of untreated pregnant women with MS (9.8 %) [28]. The background rate for spontaneous abortion is difficult to identify with precision, and spontaneous abortions were not captured prior to Registry enrollment, which may present a bias resulting in the underestimation of the true spontaneous abortion rate. However, this ascertainment bias is common to both drug exposure and general population pregnancy registries insofar as spontaneous abortions are most often reported among recognized pregnancies.

Upon review of Registry pregnancy outcomes, the Advisory Committee determined that 16 infants among the 317 (316 live births plus 1 elective termination) had defects that met criteria for major defects. The Registry birth defect rate of 5.05 %, as determined by the Advisory Committee, was higher than the 2.67 % published in the MACDP [25]. No pattern of defects suggestive of an unusual distribution was observed. Because birth outcome reference information specific to untreated MS or CD patient populations is not available, the MACDP was used as an external reference group. However, use of the MACDP as an external reference group is not without limitations. The MACDP registers birth outcomes of ≥20 weeks’ gestation with birth defects in metropolitan Atlanta; therefore, any birth outcomes of <20 weeks’ gestation are not captured. Additionally, unlike the Registry, the MACDP is not disease specific, comprises both healthy and unhealthy pregnancies, and does not target particular exposures. In contrast, the Registry population was a carefully monitored population of patients with MS or CD, which may have increased potential selection and ascertainment bias.

Registry outcomes can be complicated by potential biases inherent to the study population and design. Such biases may result in an overestimate or an underestimate of the reported risk. For example, the protocol permitted enrollment of pregnant patients in the Registry after a prenatal test, as long as testing did not indicate an abnormality. However, this practice could potentially bias the results by lowering the overall risks of birth defects [39]. Other potential factors that might confound pregnancy outcomes include maternal obstetrical history; comorbid medical conditions and medications [40, 41]; lifestyle factors (e.g., smoking or alcohol intake) [42]; and planned versus unplanned pregnancy [43]. Some caveats to consider when interpreting the Registry outcomes include differences in natalizumab exposure duration, differences in pregnancy outcome detection between the Registry and MACDP, and the absence of a comparison group of pregnant women with MS who were not exposed to natalizumab. As noted by the expert Advisory Committee, there is currently no valid and stable estimate of spontaneous abortions or major birth defects among women with MS not treated with natalizumab. In addition, previous efforts to include a comparator arm within an MS treatment pregnancy study have met with enrollment difficulties [44]. Thus, it is difficult to determine whether the outcomes observed in this Registry reflected the effects of natalizumab, underlying MS disease, or other unmeasured conditions.

## Conclusions

The Registry prospectively evaluated pregnancy outcomes in patients treated with natalizumab within 3 months of conception or during pregnancy. The overall rate of major birth defects in the Registry was higher than that reported by the MACDP. No specific pattern of birth defects was observed that would suggest a drug effect. The rate of spontaneous abortions was consistent with the expected background rates observed in the general population [26–28]. Although not observed in the Registry, one report has described transient hematological abnormalities in infants exposed to natalizumab during the third trimester [38]. The natalizumab prescribing information indicates that natalizumab should be used during pregnancy only if the potential benefit justifies the potential risk to the fetus [6]; although the Registry has noted limitations and more research is needed, the findings from the Registry may be informative to clinicians and patients in weighing potential risks and benefits of natalizumab exposure during pregnancy.

## Abbreviations

APGAR: Appearance, pulse, grimace, activity, and respiration
CC: Coordinating center
CD INFORM: Crohn’s disease investigating Natalizumab through Further Observational Research and Monitoring
CD: Crohn’s disease
MACDP: Metropolitan Atlanta Congenital Defects Program
MS: Multiple sclerosis
ROW: Rest of world
RRMS: Relapsing-remitting multiple sclerosis
TPER: Tysbari Pregnancy Exposure Registry
TYGRIS: Tysabri Global Observational Program in Safety
